# Herbal bathing: an analysis of variation in plant use among Saramaccan and Aucan Maroons in Suriname

**DOI:** 10.1186/s13002-018-0216-9

**Published:** 2018-03-15

**Authors:** Charlotte I. E. A. van ‘t Klooster, Vinije Haabo, Sofie Ruysschaert, Tessa Vossen, Tinde R. van Andel

**Affiliations:** 1 Department of Public Health and Primary Care, Leiden University Medical Center, PO Box 9600, 2300 RC Leiden, The Netherlands; 2 Saramaka Project, Kennedyweg 92, 6708 HB Wageningen, The Netherlands; 3 World Wildlife Fund Guianas, Henk Arronstraat 63, Suite E, Paramaribo, Suriname; 4 ICLON, University Leiden, Willem Einthoven Building, Wassenaarseweg 62a, 2333 AL Leiden, The Netherlands; 5 Department of Plant sciences, Subdivision Biosystematics, Wageningen University and Research (WUR), PO Box 16, 6700 AA Wageningen, The Netherlands; 6 Naturalis Biodiversity Center, Vondellaan 55, 2332 AA Leiden, The Netherlands

**Keywords:** Herbal baths, Medicinal plants, Suriname, Maroons, Saramaccan, Aucan, Traditional knowledge, Traditional medicine

## Abstract

**Background:**

Herbal baths play an important role in the traditional health care of Maroons living in the interior of Suriname. However, little is known on the differences in plant ingredients used among and within the Maroon groups. We compared plant use in herbal baths documented for Saramaccan and Aucan Maroons, to see whether similarity in species was related to bath type, ethnic group, or geographical location. We hypothesized that because of their dissimilar cultural background, they used different species for the same type of bath. We assumed, however, that plants used in genital baths were more similar, as certain plant ingredients (e.g., essential oils), are preferred in these baths.

**Methods:**

We compiled a database from published and unpublished sources on herbal bath ingredients and constructed a presence/absence matrix per bath type and study site. To assess similarity in plant use among and within Saramaccan and Aucan communities, we performed three Detrended Correspondence Analyses on species level and the Jaccard Similarity Index to quantify similarity in bath ingredients.

**Results:**

We recorded 349 plants used in six commonly used bath types: baby strength, adult strength, skin diseases, respiratory ailments, genital steam baths, and spiritual issues. Our results showed a large variation in plant ingredients among the Saramaccan and Aucans and little similarity between Saramaccans and Aucans, even for the same type of baths. Plant ingredients for baby baths and genital baths shared more species than the others. Even within the Saramaccan community, plant ingredients were stronger associated with location than with bath type.

**Conclusions:**

Plant use in bathing was strongly influenced by study site and then by ethnicity, but less by bath type. As Maroons escaped from different plantations and developed their ethnomedicinal practices in isolation, there has been little exchange in ethnobotanical knowledge after the seventeenth century between ethnic groups. Care should be taken in extrapolating plant use data collected from one location to a whole ethnic community. Maroon plant use deserves more scientific attention, especially now as there are indications that traditional knowledge is disappearing.

**Electronic supplementary material:**

The online version of this article (10.1186/s13002-018-0216-9) contains supplementary material, which is available to authorized users.

## Background

Since ancient times, people have believed that bathing in a spring, sea, or river resulted in physical and spiritual purification and thereby in the improvement of one’s health. The ancient Greeks thought that certain natural springs or tidal pools were blessed by the gods and bathing in them would help to cure diseases [1]. In Christian baptism, pouring water is regarded as a symbol of transition and renewal in which a person makes the passage from physiological birth to social birth [2]. Early descriptions (297 AD) on Japanese culture refer to ritual baths after funerals for cleansing and purification [3]. Bathing can have many different meanings across cultures, varying from an individual act concerned with cleanliness and hygiene to social acts related to rituals of purification and separation, or as a form of therapeutic practice [2].

Ethnobotanical studies have reported on bathing as a form of medical treatment in many cultures worldwide, such as Africa [4, 5], Asia [6–8], Europe [9], South America [10, 11], and the Caribbean [12–14]. Herbal baths seem to promote not only people’s physical health but also their psychological well-being. However, people’s motivations for the inclusion of certain plant species in specific types of herbal baths and regional variation in herbal ingredients remain largely understudied.

Herbal baths form a major part of the traditional medical practices of the Maroons, descendants of escaped African slaves who fought for their freedom and settled themselves in the tropical rainforest of Suriname [14, 15]. Taken from different parts of Africa, their ancestors came from numerous ethnic and linguistic groups [16]. Between 1658 and 1825, slave traders brought Africans to Suriname from many different regions and ethnicities in West and Central Africa, such as the Slave Coast (eastern Ghana to Benin), the Loango area (southern Gabon to northern Angola), Gold Coast (Ghana), and the Windward Coast (Ivory Coast, Liberia, and Sierra Leone) [17, 18]. As a result of their distinct geographical origins, cultures, and languages, the Surinamese slaves formed a heterogeneous group [18]. They brought along their own cultural heritage with values, knowledge, and beliefs to the New World, where they became a new community and began to share a culture they themselves created [16]. Since no names or origin of the slaves were registered upon arrival, linking the Surinamese Maroons to their African heritage remains difficult [19].

**Fig. 1:**
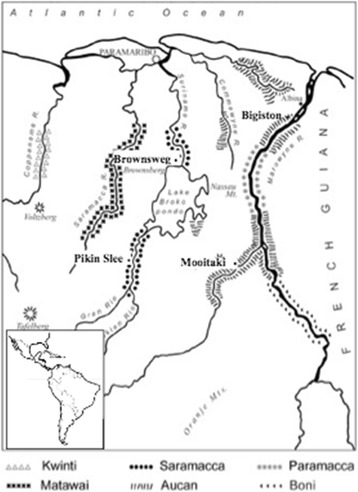
Map of Suriname, with Maroon ethnic groups and research areas. Illustration by H. Rypkema, adapted from Vant Klooster et al. [24]

Nowadays, six Maroon ethnic groups exist in Suriname (Fig. 1) with an estimated population of 127,000, which is 23% of the total population in the country [20]. The Saramaccans (pop. 58,000) and the Aucans (pop. 56,000) form the largest groups. Historically, these groups have not maintained extensive social contact with each other, as they settled among different major rivers separated by dense rainforest [21, 22]. They have lived quite isolated for centuries, which resulted in the development of their own cultures with own distinct languages (Saramaccan and Aucan), diet patterns, and traditions [23].

Maroons are well known for their traditional medicine, which usually contain a mixture of plant ingredients. Plant species used in Saramaccan and Aucan Maroon herbal baths have been the subject of recent ethnobotanical studies [12, 15, 25, 26]. Even Maroons, who migrated to The Netherlands after Suriname became independent in 1975, claimed that herbal baths were essential for their well-being and part of their cultural identity [27]. The variation of herbal baths and their plant ingredients among and within their different Maroon groups have not been studied in detail. Because most studies were conducted in a single Maroon community and hardly any data exists for the smaller Maroons groups, it is not clear how representative these studies are for Surinamese Maroons in general.

In this study, we compared plant use in six commonly applied herbal baths that were documented among the Saramaccan and/or Aucan Maroons: baths for adult strength [14, 28, 29], baby strength [13, 14, 26, 28], skin disorders [14, 28, 29], respiratory ailments [14, 28, 29], spiritual ailments [14, 15, 28–30], and genital steam baths [12, 14, 28]. In these studies, the baths were mostly described for one specific Maroon community, but no comparison was made among different Maroon groups.

The ancestors of Saramaccan and Aucan Maroons escaped from different plantations, owned by either English or Dutch masters (Aucans) or Portuguese Jews (Saramaccans), in different periods of time. The Saramaccan community developed around 1690–1710, followed by the Aucan community after 1712 [31, 32]. Their geographic separation and limited contact have probably led to distinct ethnobotanical practices. To investigate these differences, our research focused on four main questions: (1) How are the six herbal baths prepared and by whom? (2) Which plant species are used in these herbal baths? (3) How do these plant species vary between the Aucan and Saramaccan Maroons and within these groups? (4) Is similarity in plant use related to the type of herbal bath, ethnic group or geographical location?

We hypothesized that Aucan and Saramaccan Maroons used different plant species for the same type of herbal baths. We anticipated that especially spiritual baths would show a low similarity in plant species, based on the symbolic meaning of the most plant ingredients, which is more related to specific Maroon cultural histories than to pharmacological content [15, 25]. However, we also hypothesized that plants used in genital steam baths would show a larger similarity, based on the selection of plant ingredients containing certain active substances preferred in these baths, such as tannins and essential oils [12, 25]. We expected differences in plant species to be determined by the type of bath (application) and by ethnicity (cultural preference). Finally, we expected to find the most overlap in plant species for the genital baths within the Saramaccan community.

The outcome of this research will contribute to the overall knowledge of traditional health care practices among the Aucan and Saramaccan Maroons and could form a basis for further assessment of the medical effectiveness of bath treatments and the therapeutic potential of medicinal plants in general. Analyzing the variation of Maroon traditional medical knowledge will not only contribute to a better understanding of their cultures and their strong relationship with nature but also verify how representative plant use documented in a specific village is for the entire ethnic group. Our outcomes will clarify whether plant use within an ethnic group is uniform or variable.

## Methods

### Data collection

Data on plant species used in herbal bath treatments were retrieved from published ethnobotanical fieldwork studies among Suriname Maroons [12–15, 25, 26, 29], and the original databases on which these studies were based, as these contain extra information on herbal baths (Table 1). We also added data from unpublished student reports [28, 30].

**Table 1:** Sources of ethnobotanical data used in the analysis

Ethnic group	Location fieldwork	Year	Data source	Nr. of species in herbal baths	Nr. of respondents	Publication based on fieldwork
Saramaccan	Brownsweg (Brokopondo Lake)	2005–2006	Fieldwork Ruysschaert	274	ca. 200	[13, 15, 25, 29]
Saramaccan	Pikin Slee (Upper Suriname River)	2009	Fieldwork Van’t Klooster	69	20	[14, 28]
Aucan	Bigiston (Marowijne River)	2006	Fieldwork Van Andel	87	3	[12, 15, 25, 30]
Aucan	Mooitaki (Tapanahoni River)	2013	Fieldwork Van Andel and Vossen	39	25	[26]

During these studies, information was gathered through semi-structured interviews and plant collection trips. Research and collection permits were obtained from the Surinamese Forest Service department (LBB) and oral or written prior informed consent from participants and the respective village authorities. The purpose and nature of the research were explained to respondents before the interviews took place. All studies from which we used data followed the Code of Ethics of the International Society of Ethnobiology [33]. Respondents knowledgeable on medicinal plants and their uses were identified through previous contact with community members and snowball sampling and included laymen and traditional healers, males and females, and young and older people. Other methods used were informal (group) conversations, free listing, participant observations, household surveys, and assignment techniques [34]. Topics included plants used for health promotion, disease prevention and cure, their growth forms, local names, parts used, mode of preparation, route of administration, health concerns, and knowledge transfer.

Interview questions were pre-tested with the help of bilingual Surinamese key informants to check whether the questions were framed in correct Sranantongo, Saramaccan, or Aucan and pertinent to the research. Some interviews were recorded with a voice recorder and transcribed in Saramaccan to be discussed with informants to verify answers. Plant species not immediately familiar to the researchers were collected as botanical vouchers and identified and deposited at the National Herbarium of Suriname (BBS, all specimens), Naturalis Biodiversity Center (L, except the Brownsweg and Pikin Slee collections), and Ghent University (GENT, Brownsweg collections only). The definitions of the different herbal baths were documented during fieldwork and afterwards checked with the key informants for correct interpretation.

### Data analysis

Since most data was collected in the Saramaccan area, we selected herbal baths that were frequently used at the two Saramaccan locations (Brownsweg and Pikin Slee), and added the available data of the two Aucan locations (Mooitaki and Bigiston) for comparison. Each bath type should at least be used at two out of the four study sites to allow comparison. All plant species used in the six selected herbal bath types (baby strength, adult strength, genital steam baths, skin disorders, respiratory ailments and spiritual ailments) are listed in Additional file 1 with corresponding information on vernacular names, scientific names, family, and collection numbers (when available).

We constructed a presence/absence matrix in Excel with all recorded plant species in rows and the type of herbal bath and the four different Maroon villages as columns. We entered a 1 in the cells when a species was used in a particular bath type and a 0 if the species was not used in that type of bath (Additional file 2). All plants used in a certain herbal bath type (per location) were used as the sample unit in our analysis. Unidentified plants were excluded from our analyses. All scientific names were checked and updated by means of The Plant List [35]. All Maroon plant names were checked and updated following the latest spelling rules [24, 25, 36].

To quantify similarity in herbal bath ingredients among the two Maroon groups (Saramaccan versus Aucan) and within these ethnic groups on village level, we utilized the Jaccard Similarity Index [37]. This index is based on plant presence or absence in a community or in data sets, while relating the number of species in common with respect to the total number of species, expressed as JI = (*c*/*a* + *b* + *c*), where *c* is the number of species in common, *a* is the number of unique species of community A, and *b* is the number of species solely of community B. Similarity coefficients vary from a minimum of 0 (when the communities do not share any species) to 1 (when all plants used are identical). The outcome is often multiplied by 100 to obtain percentages.

To visualize the similarity in plant use between our Saramaccan and Aucan Maroon study sites and the six herbal baths types, we performed a Detrended Correspondence Analysis (DCA) on species level [38]. We plotted the results of our DCA on the two main axes that caused the distribution of the data to visualize potential overlap and variation in plant use. To compare plant use within the Saramaccan population at the locations Pikin Slee and Brownsweg, we plotted the results of another DCA analysis (with Saramaccan data only) to visualize potential overlap and variation in herbal bath ingredients. Finally, we plotted the results of a third DCA analysis to visualize overlap and variation among all four locations. All DCA analyses were performed in the program PC-ORD version 5.32.

To see whether differences in plant use between the two Maroon groups could be caused by differences in the occurrence in plant species between the study sites, we first checked the ethnobotanical literature whether the species were used for other purposes by both Maroon groups, and if not the case, whether local names existed for these species in both Aucan and Saramaccan languages [24], as this indicates species’ occurrence in the specific Maroon territory. For species for which no ethnobotanical information existed for either Saramaccans or Aucans, we checked the geographical distribution in the floristic literature of Suriname and the Guianas [39–42] and online collection databases [43] to see whether they had restricted, patchy, or wide distributions.

## Results

### Herbal baths

Maroons use herbal baths for both physical and spiritual cleansing. They form an important aspect of their cultural practices and daily life. Some herbal baths are made for one person, others for more people or the whole village. Herbal bath preparations and applications may be very elaborate. It is common to have a mixture of leaves in water standing in a plastic tub or a big earthen or wooden dish in front of the house for days to be used many times by one or more family members by adding new water to it before pouring the mixture over the head and/or body with a calabash (*Crescentia cujete*). Aromatic plant species, such as *Campomanesia aromatica* and *Lantana camara*, are often added to baths for overall body refreshment and their agreeable smell.

Herbal baths are mostly taken in the village, but also in the forest, at the riverbank or in the river itself. They can include the use of rum, kaolin (*pemba*), magical objects, and spiritual sayings. Baths are prepared by male and female laymen for family use only but also by herbalists and spiritual healers. The latter are believed to possess magical powers and use ritual instruments to contact the spiritual world to find answers to the problems perceived. A spiritual healer (*obiaman*) is often consulted when self-treatment or biomedical cures do not have the desired result. Spiritual healers should be paid in the form of goods (*madyomina*), such as pieces of cloth, soft drinks, or other valuables, to make the herbal bath work. Although this compensation system still exists, healers increasingly request cash payment for their services.

#### Adult strength

**Fig. 2:**
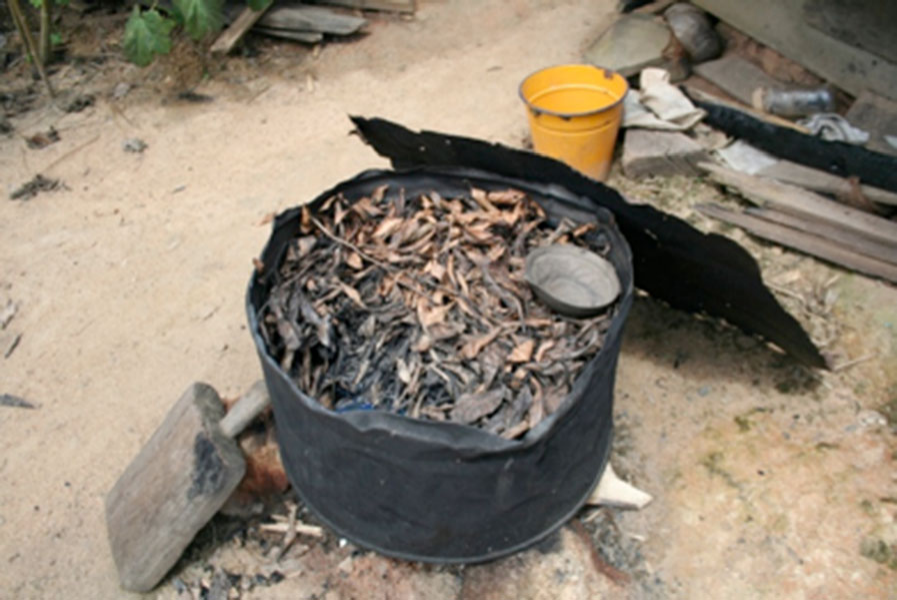
Saramaccan bath for adult strengthening, Pikin Slee, 2009. Picture by C. van’t Klooster

For Maroons, it is important to keep the body healthy and strong. Aucans refer to the strengthening baths as *taanga sikin uwii* and the Saramaccans as *taanga sinkii uwii*. These baths are used to give physical strength (*taanga* from “strong”) to the body (*sinkii* from “skin/body”), by the use of different plant leaves (*uwii* from “leaves/weed”). The treatment is also taken when people feel tired or suffer from backaches and other body pains due to heavy physical work. Men prepare these baths mainly at the start of intensive manual labor, like cutting new fields for their wives or making a new boat. Women, who are responsible for maintaining and harvesting the fields, use these strengthening baths on a regular or even daily base. Baths are often prepared in house yards for family use, by adding different plants (twigs and leaves of trees and lianas, shrubs, and herbs) to a metal barrel filled with water placed on a wood fire to heat up before use (Fig. 2). The mixture is poured on top of the head with a calabash, often leaving remnants of leaves on the body, which should not be washed away for the medicine to work properly. No data have yet been collected on the plant ingredients of this specific bath among Aucans.

#### Baby strength

Newborns are frequently bathed to improve their strength, to stimulate them being active and curious, and to start crawling and walking early. Mothers find this essential to be able to conduct their own daily activities. These baby baths, also known among Maroons as “waka snel” (walk early), are prepared by boiling plants in small pots after which the hot decoction is diluted with cold water to make it lukewarm. The ingredients may be boiled again for several days, before the plants are replaced by fresh ones. Baby baths contain other plant species than strengthening baths for adults. They are used until the child is old enough to bathe by itself.

#### Respiratory ailments and skin disorders

Physical ailments such as cough, colds, and headaches due to blocked sinuses are treated by taking a facial steam bath, prepared by boiling a mixture of herbs in water. The steam is inhaled while hanging over a bowl with a herbal decoction; the head is covered with a piece of fabric. Some patients take this bath while covering themselves completely with a sheet bending over a large bucket. Sometimes, a whole house is filled with steam, like a sauna. The Saramaccans refer to this type of steam bathing as *suwa uwii* (“herbs for steaming/sweating”). Another type of herbal bath is used to treat skin conditions, varying from pimples, eczema, and psoriasis to fungal infections. These baths, to which the Saramaccans refer as *bita* (“bitter”), consist of a single plant or a mixture of species with a bitter taste. The plants are boiled in water in small pots on the fire, after which the cooled decoction is applied to the body with a calabash. No records exist for the Aucans on these two types of herbal baths.

#### Genital steam baths

A very frequently used herbal bath among Maroons is the genital steam bath. Women use plant decoctions to clean, heal, refresh but also tighten the vagina, or to enhance sexual pleasure. They prepare this bath in their backyard kitchens, which are separated from their main house. Plants are added to water and boiled. Women sit on a bucket and let the steam enter their vagina; afterwards, they wash their genitals with the somewhat cooled decoction. Such cleansing baths are often taken on a daily base (in the morning), while baths to enhance recovery after delivery are taken in the first few weeks after giving birth. Saramaccans refer to these baths as *kete uwii* (“*kete*” from kettle, *uwii* from weed), *muyee uwii* (“*muyee*” from woman), or *wasiwoyo uwii* (vaginal wash). Aucans know this genital bath also as *ketee uwii* and under the names *gogo uwii* (“bottom herbs”) or *uma patu* (“*uma*” from woman, “*patu*” from pot).

#### Spiritual baths

**Fig. 3:**
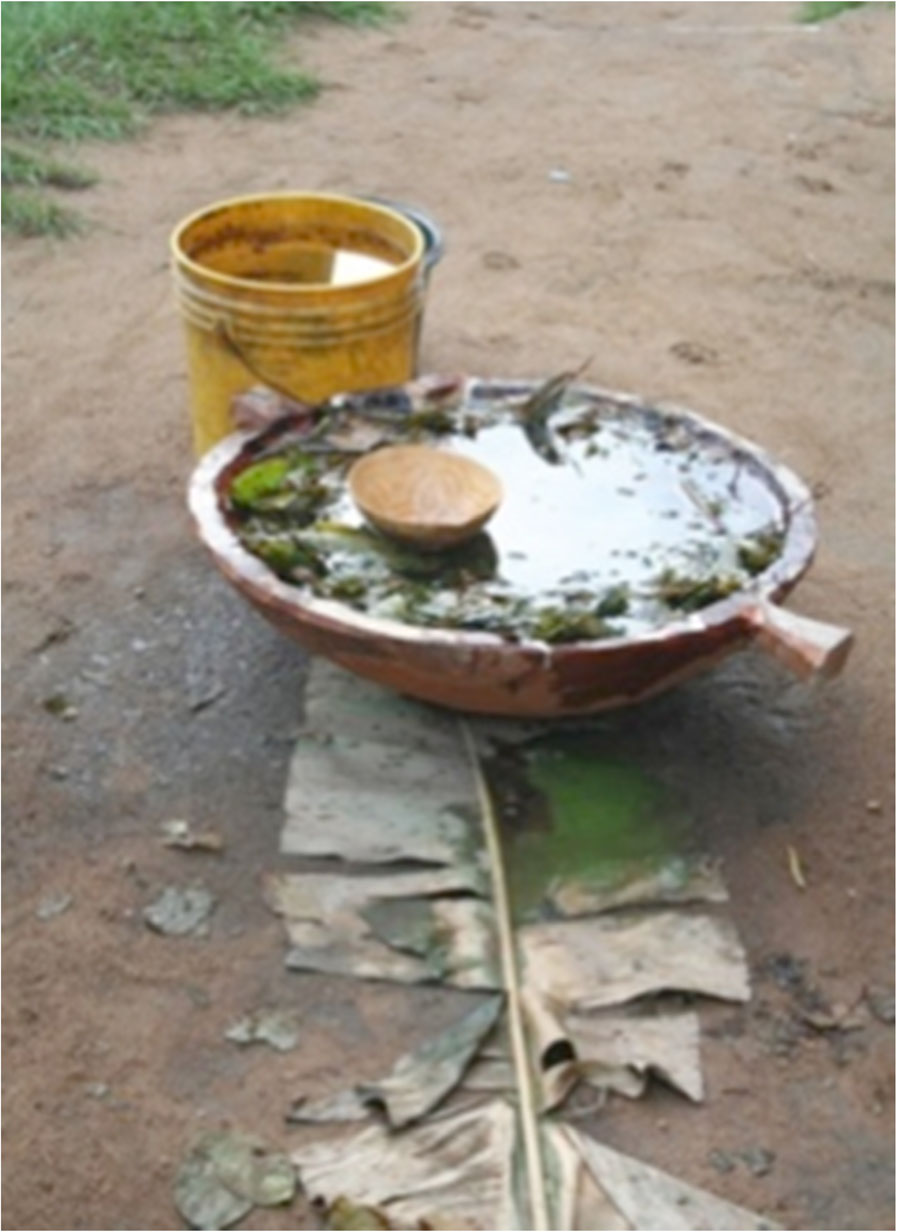
Spiritual herbal bath *limbo baka*, placed on a banana leaf, Pikin Slee 2009. Picture by C. Van ‘t Klooster

In Maroon cultures, the spiritual world takes an important place in daily life. When an ailment cannot be cured by modern medicine or by self-prepared home medicines, it is said to have a supernatural cause, such as agitated water or forest spirits, ancestral spirits, or witchcraft. In these cases, a spiritual healer or experienced family member needs to be consulted, who will reveal the supernatural cause and treat the person accordingly, often by a specific herbal bath. Spiritual baths are generally referred to “*winti wasi*” (Aucan) or “*obia uwii wasi*” or “*gadu uwii wasi*” (Saramaccan). Spiritual baths have different names according to the causes targeted and type of spirits or gods (*gadu*) and magical powers (*obia*) involved. The herbal bath *limbo baka* (Saramaccan) is used to ward off evil, such as a *yooka* (malicious spirit of a deceased person) and magical poisons made and sent by people to harm someone. It is also used as protection to secure a safe travel or to attract luck or material welfare. *Limbo baka* literally means cleaning (*limbo*) one’s back (*baka*) from bad spirits. This treatment can be taken individually or together with family members. Saramaccan healers make this bath in a big earthen or wooden dish (Fig. 3).

Persons who take this bath should stand barefoot on a banana leaf (*Musa* sp.), facing east for the best result. Circles of kaolin are sometimes drawn around the herbal bath to keep evil spirits out and show other villagers that this bath is related to *obia* (magical powers) or nature spirits like the air god *Komanti*. While taking the bath, the person has to apply water on the head and body with a small calabash. The bathing person and/or healer prays to the highest God, ancestral spirits, and other gods to request for luck, good health, and guidance. Bathing is often combined with spitting rum or water in different directions around the person. Another bath is *wasi gadu* (“wash gods”), which is often taking place on village level to ward off evil spirits or to honor good ones, frequently combined with ritual songs, prayers, and offerings. Every village, family and related clan (“*lo*”) has its own *gadu* that they need to take good care of. *Paati wasi* (Sa) is a spiritual bath used to release a spirit that has a negative effect on one’s well-being. It can also eliminate bad feelings caused by a traumatic experience. This spirit is often recognized as a deceased family member, not necessarily of bad intentions. If it affects the person negatively, it needs to be separated (*paati*) from the person by taking the herbal bath on a crossroad in a forest trail. Both the *paati wasi* and *limbo baka* treatments (on family level) can be finalized in the river with an additional washing, during which persons are hit on their back with a bundle of leaves to expel the spirits from their bodies.

### Most widely used plant species

In total, 349 plant species were used in the six herbal bath types. Table 2 shows the amount of species used per bath type for the different study sites. Due to the lack of data for Aucan baths, a comparison could not be made for all types.

**Table 2:** Number of plant species used per type of herbal bath, Maroon group and study site

Bath type	Saramaccan			Aucan		
	Pikin Slee	Brownsweg	Total	Bigiston	Mooitaki	Total
Baby strength	26	101	116	–	33	33
Genital	26	149	160	45	–	45
Respiratory	16	49	56	–	–	–
Skin disorders	8	38	43	–	–	–
Spiritual	17	151	155	49	12	59
Strength adults	21	81	95	–	–	–
Total number of plant species per location*	69	274	302	87	39	115

*As species can be used in several types of herbal baths, this number is smaller than the sum of the species numbers for each bath type

Of the 302 herbal bath species recorded among the Saramaccans, 12 were used in all of the six bath types (*Cecropia sciadophylla*, *Chromolaena odorata*, *Commelina erecta*, *Gossypium barbadense*, *Lippia alba*, *Lantana camara*, *Rolandra fruticosa*, *Siparuna guianensis*, *Stachytarpheta* *cayennensis*, *S. jamaicensis*, *Tilesia baccata*, and *Unxia camphorata*). With exception of the cultivated *G. barbadense*, all these species are common in secondary vegetation. Thirteen species were used in five baths, 21 in four baths, 66 in two baths and 149 in just one type of bath. This means that 51% of the species (153 spp.) were used in more than one bath type. The fact that most species were recorded in Brownsweg is probably a result of a larger research effort (e.g., time spent in village, data sampling design), a stronger research focus on bathing, and the much larger population size in comparison with the three other study sites.

Out of the 115 plant species used by Aucans, only *Cyperus prolixus* was used in all three types of baths (baby strength, genital and spiritual). A total of 20 (17%) species were applied in two baths, while 94 species were used in a single bath type. The higher number of species recorded for Bigiston can be explained by the fact that the research in Bigiston focused more on ritual practices, while that in Mooitaki on baby baths.

A number of 68 plant species (19% of the total) were used by both Maroon groups, of which 58 (16%) for the same type of herbal bath (Table 3). *Abuta grandifolia*, *Ertela trifolia*, *Inga stipularis*, *Ischnosiphon gracilis*, *Lueheopsis rosea*, *Nepsera aquatica*, *Philodendron hederaceum*, *P. perrottetii*, *Phthirusa stelis*, and *Tripogandra serrulata* were commonly used in herbal baths by both Saramaccans and Aucans, but not for the same bath type.

**Table 3:** Plant species used by both Saramaccan and Aucan Maroons in the same type of bathing

Genital bath (28 spp.)	Spiritual bath (20 spp.)	Baby strength (17 spp.)
Anacardium occidentale*	*Begonia glabra*	Arachis hypogaea*
*Campomanesia aromatica*	*Campomanesia grandiflora*	*Campomanesia aromatica*
*Campomanesia grandiflora*	*Chromolaena odorata*	*Chromolaena odorata*
*Cecropia obtusa*	*Costus scaber*	*Eleusine indica*
*Cecropia sciadophylla*	*Cyathula prostrata*	Gossypium barbadense*
Citrus aurantiifolia*	*Eclipta prostrata*	*Handroanthus serratifolius*
*Clidemia hirta*	*Eleusine indica*	*Hiraea faginea*
*Copaifera guyanensis*	*Euphorbia thymifolia*	*Ischnosiphon puberulus*
*Cordia schomburgkii*	*Heliotropium indicum*	*Oryctanthus florulentus*
*Davilla kunthii*	Indigofera suffructicosa*	*Paspalum conjugatum*
*Eclipta prostrata*	*Ischnosiphon arouma*	*Paullinia pinnata*
*Euphorbia hirta*	*Justicia pectoralis*	*Portulaca oleracea*
Gossypium barbadense*	Ocimum campechianum*	*Rolandra fruticosa*
Inga edulis*	*Peperomia pellucida*	*Stachytarpheta cayennensis*
*Lantana camara*	*Piper bartlingianum*	*Trema micrantha*
Lippia alba*	*Scoparia dulcis*	*Vismia macrophylla*
Mangifera indica*	*Selaginella parkeri*	*Vouarana guianensis*
*Marlierea montana*	*Senna quinquangulata*	
*Melaleuca cajuputi*	*Stachytarpheta cayennensis*	
*Miconia lepidota*	*Uncaria guianensis*	
*Myrciaria floribunda*		
*Piper arboreum*		
Piper marginatum*		
Psidium guajava*		
*Siparuna guianensis*		
Syzygium cumini*		
*Vismia cayennensis*		
*Vismia guianensis*		

*Domesticated and cultivated species

### Similarity in plant use

The similarity in plant use between the two Maroon groups, within the Saramaccan group (Brownsweg versus Pikin Slee) and within the Aucan group (Mooitaki versus Bigiston), is presented in Table 4. Generally, there was a very low similarity in plant use, varying between 3 and 16%. Saramaccan baths for respiratory ailments showed the highest similarity in plant ingredients and often contained fragrant species such as *Citrus aurantiifolia*, *Cymbopogon citratus*, *Lantana camara*, and *Siparuna guianensis*.

**Table 4:** Jaccard similarity coefficient to show the similarity in plant use between the Saramaccan and Aucan Maroons and within the Saramaccan and Aucan population

Bath type	Saramaccan	Aucan	Saramaccan versus Aucan
	Pikin Slee versus Brownsweg	Bigiston versus Mooitaki	
Baby strength	0.09		0.13
Genital	0.09		0.12
Respiratory	0.16		
Skin disorders	0.07		
Spiritual	0.08	0.03	0.10
Strength adults	0.07		

**Fig. 4:**
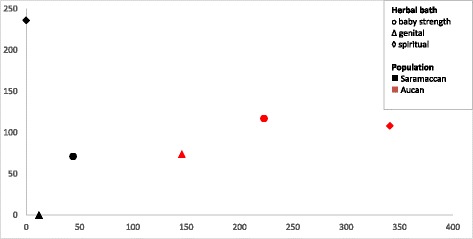
DCA scatterplot showing similarity in bath use on species level (*n* = 309). Data points indicate plant species used in specific baths by the two Maroon groups. The closer the data points, the greater similarity in plant species**.** Axes do not represent variables but serve to visualize variation and similarity in plant use

When we plot the results of our DCA analysis, carried out for the three baths shared by the two Maroon groups, we see that plant species cluster by ethnicity rather than by type of herbal bath (Fig. 4). For both Maroon groups, the genital and the baby baths are more similar in plant species than the spiritual baths, which confirm the results of the Jaccard index.

**Fig. 5:**
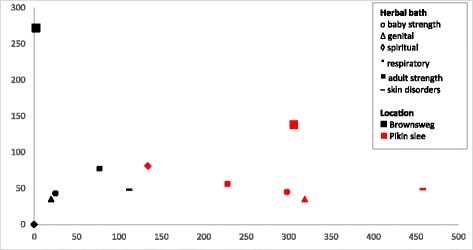
DCA scatterplot showing similarity in herbal bath type use on species level (*n* = 302). Data points indicate plant species used in specific baths by Saramaccan Maroons at two different locations. Clustered data points indicate similarity in plant species used. Axes do not represent variables but serve to visualize variation and similarity in plant use

When the herbal bath species are compared within the Saramaccan population, we see considerable differences between the locations Pikin Slee and Brownsweg for the six types of baths they have in common (Fig. 5). All the baths for Brownsweg seem to cluster to the left while the baths for Pikin Slee seem to cluster to the right side in the figure. Baby baths and genital baths are more similar in species composition than other baths.

**Fig. 6:**
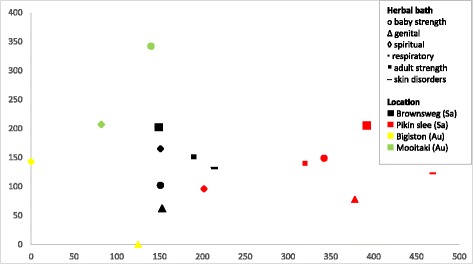
DCA scatterplot showing similarity in herbal bath type use on species level (*n* = 349). Data points indicate plant species used in specific baths by Saramaccans and Aucans at four locations. Clustered data points indicate similarity in plant species used

To determine the similarities in plant use between the Maroon locations separately, we plotted the baths for all four study sites (Fig. 6). Again, plants clustered according to geographical location rather than per herbal bath, and to a lesser extent by ethnic group, as all Aucan baths group to the left and all Saramaccan baths to the right. This could indicate that each Maroon community (study site) has adapted its plant use to the species that were locally available, based on knowledge generated over centuries of relative isolation.

When we compared our data with literature on Saramaccan and Aucan plant uses, we found that apart from the 68 shared bath species, 177 spp. (51%) of the plant species used in our analysis were used by both Maroon groups for a variety of purposes, indicating species occurrence in both locations (Additional file 1). For the remaining 172 species, no additional Maroon vernacular names or plant uses were found to prove their existence in both areas. However, 29 of these species were common weeds or secondary forest plants, eight were common forest species and eight were known to be widely cultivated (e.g., *Terminalia catappa* and *Syzygium malaccense*), mostly known by their Sranantongo names, so these species have a high probability to occur in both Maroon areas. This would make it plausible that in total 64% (222 spp.) would occur in both places. Of the remaining 127 species, 35 spp. were not identified to species level or we had doubts about their identification by others, so comparison was not possible. For the residual 92 species, 15 species grow mostly in savanna areas that are close to the Saramaccan settlements, but far away from Aucan villages and three species (*Ocimum gratissimum*, *Hymenocallis tubiflora*, and *Rhizophora racemosa*) were reported from Aucan areas only. The remaining 74 species (21%) were uncommon rainforest species (e.g., *Diospyros* cf. *cavalcantei*, *Pouteria engleri*) with patchy distributions. Due to the lack of detailed species distribution maps for rainforest trees in the interior of Suriname we could not verify whether these species occurred in both Maroon areas.

## Discussion

### Variation in plant use among the Aucan and Saramaccan Maroons

Our results show a large variation in plant use for herbal bathing among the four Maroon communities. This is partly in line with our hypothesis that plant use differs among Maroon groups as they had little opportunity for ethnobotanical exchange in the past centuries. We expected to find more similarity in genital baths, assuming that these plants would be selected on their pharmacological effect instead of their supposed symbolic or cultural meaning. This was the case to a certain extent (a similarity of 12%), as many of these baths contained species with essential oils (e.g., *Campomanesia aromatica*). Therefore, our hypothesis should be accepted, although the species overlap in baby baths was slightly higher (13%). Plant use was more strongly associated to ethnicity (Aucan or Saramaccan) than to type of bath. Both Maroon groups seem to use the same plant species for different treatments. For example, *Eclipa prostrata* is used by Aucans in bath for protection against bullets, while Saramaccans use it together with *Attalea maripa* in body scarification for esthetic purposes [25].

The difference in plant use between Saramaccan and Aucan Maroons could be explained by the fact that their forefathers escaped from different plantations, with either, English, Dutch, or Portuguese Jewish plantation owners, in different periods of time. While the Saramaccan community developed around 1690–1710 during the second wave of Maroons escaping the Portuguese Jewish plantations, the Aucan community was formed only after 1712, when Suriname was attacked by French admiral Cassard, which led to a new wave of runaways [31, 32]. The two Maroon groups have settled along different rivers in the tropical rainforest, where they had to adapt to new surroundings. For centuries, they had little contact, caused by the impenetrable stretches of forest between the rivers and by mutual distrust, and thus the exchange of ethnobotanical knowledge was limited [21, 22]. After a relative short stay at the plantations surrounded by people of mixed African origin, the runaways escaped per plantation into the interior [31]. In order to survive in the rainforests full of unknown species, Maroons had to rely largely on their own African knowledge to survive with little opportunities to exchange information with indigenous and other Maroon groups [22].

Van Andel et al. [38] showed in their study on plants used in bitter tonics across the Atlantic that enslaved Africans in the New World had to reinvent their aphrodisiac mixtures in their new living environment. They used their knowledge and the local flora that was available to them. Enslaved Africans in Suriname searched for similar plants that they remembered from their homeland, but succeeded in this only for a small number of plant species. Instead of limiting themselves to their previously known plant species, they searched for new species to replace the African ingredients in their herbal treatments [26]. This is also illustrated by the fact that Maroons have used many African plant names to name botanically related species in Suriname [18].

A recent study conducted by Tareau et al. [44] on medicinal and cosmetic plants used by urban youngsters in French Guiana also showed that bathing formed an important way of administrating herbal medicine. Most of the medicinal baths reported in our study correspond with the therapeutic functions of baths reported by Tareau et al., [44]. However, baths related to adult strength were not reported for French Guiana, although there are several Saramaccan communities in that country. Probably, baths for strength seem not that important for the French Guianese youth, reflecting their life styles in more urban settings.

Herbal bath treatments seem to have evolved over time not only during the enslavement period but especially after the formation of independent communities in the isolated forests. Since the runaways that formed the current Aucan and Saramaccan communities came from different plantations, and lived in geographically isolated areas without roads, they had little opportunity to share their plant use knowledge gained during and after the period of enslavement. Therefore, the current overlap in plant species could be a result of a more recent knowledge exchange. A comparative analysis between data of Van Andel and Havinga [45] and our data showed that 69% (47 of the 68) of the plant species used by both Maroon groups are nowadays sold on markets in Paramaribo. It is plausible that exchange is currently taking place at the markets, where hundreds of Maroon women sell their herbal medicine to city-dwelling Maroons of different ethnicities. From Paramaribo, Maroons transfer their newly acquired knowledge to the forest communities when visiting their family in the hinterland. For example, the aromatic tree *Melaleuca cajuputi*, originally from Australia, was introduced to eastern Suriname via French Guiana. It has become invasive along the Marowijne River close to Albina, and is known locally as Albina uma (Au). This tree, originally planted by Catholic nuns in French Guiana for its essential oil, became popular in Suriname for its *Eucalyptus*-like scent to treat symptoms of cold and bronchitis. Nowadays, the leaves of this tree are sold in Paramaribo to use in genital steam baths for their refreshing smell. The Saramaccans also know this plant under the name Albina uma, which shows that exchange of knowledge among Maroons takes place in Paramaribo [25].

### Variation in plant use within the Saramaccan ethnic group

Our results clearly show that plant use within the Saramaccan group is also location specific, as Saramaccan bath ingredients differed substantially between the two study sites. The plant species in baby baths and genital baths showed a stronger similarity than the other baths, but overall there was little overlap between Pikin Slee and Brownsweg. Our research findings support earlier studies by Van Andel et al. [15] and Van’t Klooster et al. [14] that showed that many plant species used in herbal baths were not bath-specific, as most of them were used in more types of baths. Brownsweg and Pikin Slee are surrounded by similar tropical rainforest vegetation [46], although near Pikin Slee the primary forest is more extensive. However, since most species used occur in secondary forest or open vegetation around villages, the variation in plant use could not be justified by differences in vegetation alone.

The explanation for the dissimilarity in plant use between the two study sites can probably be found in the social structure of Maroon culture. Although the Saramaccans have a common cultural background, the Upper Suriname River area, where Pikin Slee is located, has been characterized as very traditional and the center of Saramaccan cultural traditions [47]. Not only in geographical but also in social sense, this area has been quite isolated [48]. Traffic between Brownsweg and Pikin Slee still takes several hours by bus and boat, limiting the amount of ethnobotanical knowledge exchange.

Maroon societies, with their own paramount chief and local government, are characterized by a matrilineal relationship structure with social units called matriclans (*lo’s*) and matrilineages (*bees*). While the first Saramaccan clan was formed in 1690 [49], nowadays approximately 12 clans are in existence. Their social identity, rights to land and associated resources, and social obligations are all determined by these *bees* and secondarily to these *lo’s* they belong to [50]. It was recently shown that knowledge of plant species is generally widespread, but the details of the processing and administration methods is often kept within family groups [14]. Furthermore, restrictions in plant use do exist for certain matrilineages, families or even for a whole village. This could explain the difference in plant use within the Saramaccan community and between the two Maroon groups, although the plant species themselves occur mostly in both localities.

Although a number of studies have been conducted among Maroons in neighboring countries like French Guiana [51, 52] and Brazil [53–63], publications on medicinal plant uses and knowledge held by traditional Maroon communities in primary forests are still scarce. A recent study by De Santana et al. [62] conducted among the Afro-Brazilian Salamina community living in an isolated region of Bahia, reported a considerable amount of medicinal plant knowledge. However, little plants were used from the surrounding old-growth tropical forests. The large number of cultivated exotics and weedy plants found by De Santana [62] was also reported in other publications on Maroon communities [59, 63]. Zank and Hanazaki [63] suggest that the focus on disturbance species reflects the environmental conditions and history of the Brazilian Maroon region. The Surinamese Maroons still have access to primary rainforest, although they also make use of domesticated plant species such as mango or cotton leaves and cultivate some wild species in their home gardens [45]. To get a better overview in the differences and similarities in Maroon plant use, further research should be conducted among Maroons that live in remote, forested areas in the Guianas and Brazil, using similar methods and sample efforts at all study sites. This type of research will gain more insights in shared cultural knowledge than is now possible with the scarce information currently available for Suriname. These data could then be compared to Maroon studies in deforested or urban communities in these countries.

### Limitations of this study

Because of the differences in scientific approach, data collection methods, and sampling effort at the study sites in Suriname, hard claims on shared ethnobotanical knowledge cannot be made. For such claims, it is essential that the same research methods are used at each study site so that a cultural consensus model could be developed as showed by Reyes-Garcia et al. [64]. The differences in research methods at the two Saramaccan locations may have influenced the number of recorded plant species per bath type in our comparative study. The larger sampling effort in Brownsweg probably gives a more complete representation on plant use. We are aware that our body of data on Aucan plant use is much smaller and thus less representative. The current data is therefore not sufficient to assess cultural knowledge on population level, especially for the Aucans. Data from Bigiston were mainly retrieved from one traditional healer [30], although his knowledge was quite extensive. There is a substantial group of Aucans living along the Cottica River near the coast, where no ethnobotanical research has been carried out so far. More research among Aucans would probably yield more used species and somewhat higher Jaccard indices. However, since almost half of the 115 species used by the Aucans in our study were not used by the Saramaccans, differences in plant use between the two groups are certainly visible. For the other four Maroon groups (Kwinti, Paramaccan, Boni, and Matawai) almost no data exist on medicinal plant use.

## Conclusion

Our study on herbal bathing practices and plant ingredients showed little similarity between Saramaccan and Aucan Maroon groups, even when the same bath types were examined. Plant use appeared to be strongly influenced by study site and then by ethnicity, but less by application. Due to isolation and adaptation processes, Maroon plant use kept evolving over time and space. Whether this will continue and how, depends on the level of contact and knowledge exchange between Maroon communities. Due to migration to the capital and higher accessibility of the interior, their social environment will likely continue to change in the future. Our research showed substantial differences in plant use among Maroons communities with regard to six herbal baths, but little is known on similarities in the many other herbal medicine practices among these groups. Our results further suggest that care should be taken in extrapolating plant use data collected in one location to an entire ethnic group. We hope that our study will contribute to the conservation of Maroon biocultural knowledge and will create awareness to the elaborate traditional medical practices of Surinamese Maroons.

## Additional files

Additional file 1: Plant species used in herbal bathing in Suriname with family, scientific and vernacular names. (XLSX 65 kb)

Additional file 2: Matrix with plant species used per herbal bath type per Maroon community as well as location. (XLSX 188 kb)
